# Clinical malaria among pregnant women on combined insecticide treated nets (ITNs) and intermittent preventive treatment (IPTp) with sulphadoxine-pyrimethamine in Yaounde, Cameroon

**DOI:** 10.1186/1472-6874-14-68

**Published:** 2014-05-16

**Authors:** Robinson Enow Mbu, William Ako Takang, Hortence Jeanne Fouedjio, Florent Ymele Fouelifack, Florence Ndikum Tumasang, Rebecca Tonye

**Affiliations:** 1 Department of Obstetrics and Gynecology, Faculty of Medicine and Biomedical Sciences, University of Yaounde 1, Cameroon and the Maternity Unit, Central Hospital, Yaounde, Cameroon; 2 Maternity Unit, Central Hospital Yaounde, Yaounde, Cameroon; 3 Department of Obstetrics and Gynaecology, Faculty of Health Sciences, University of Bamenda, Cameroon, Bamenda, Cameroon

**Keywords:** Clinical malaria, Intermittent preventive treatment, Insecticide treated nets

## Abstract

**Background:**

Malaria remains a burden for pregnant women and the under 5. Intermittent preventive treatment of pregnant women (IPTp) for malaria with sulfadoxine – pyrimethamine (SP) has since replaced prophylaxis and legislation has been reinforced in the area of insecticide treated mosquito nets (ITNs) in Cameroon. Clinical malaria despite all these measures remains a problem. We compared the socio-obstetrical characteristics of women who developed clinical malaria and those who did not though in the same regimen.

**Methods:**

A 5 – year nested cohort study (2007 – 2011 inclusive) at the tertiary level hospitals in Yaounde. Pregnant women who willingly accepted to participate in the study were enrolled at booking and three doses of SP were administered between 18 – 20 weeks of gestation, between 26–28 weeks and between 32 – 34 weeks. Those who developed clinical malaria were considered as cases and were compared for socio – obstetrical characteristics with those who did not. Venous blood was drawn from the women in both arms for parasite density estimation and identification and all the clinical cases were treated conventionally.

**Results:**

Each arm had 166 cases and many women who developed clinical malaria were between 15 and 19 years (OR 5.5, 95% CI 3.9 – 5.3, p < 0.001). They were of low gravidity (OR 6.5, 95% CI 3.8 – 11.3, p < 0.001) as well as low parity (OR 4.6, 95% CI 2.7 – 7.9, p < 0.001). The cases were single women (OR 4.58, 95% CI 2.54 – 8.26, p < 0.001) and had attained only primary level of education (OR 4.6, 95% CI 2.8 – 7.9, p < 0.001). Gestational ages were between 20 to 30 weeks during clinical malaria (OR 6.8, 95% CI 4.1 – 11.7, p < 0.001). The time between the first and second dose of SP was longer than ten weeks in the cases (OR 5.5, 95% CI 3.2 – 9.3, p < 0.001) and parasite density was higher also among the cases (OR 6.9, 95% CI 5.9 – 12.1, p < 0.001).

**Conclusion:**

Long spacing between the first and second dose of SP seemed to be responsible for clinical malaria in the cases.

## Background

Malaria in pregnancy (MiP) generates public health concerns across the globe, particularly in malaria – endemic settings and results in adverse outcomes both for the woman and foetus [1-5]. Malaria in Cameroon, as it is in other resource restricted countries has a negative impact on the health of children and pregnant women, especially the primigravidae. Indeed, higher susceptibility to malaria is noted among pregnant women probably due to a decrease in their immune responses [6-8].

Management of malaria in pregnancy now combines multiple approaches [9-11]. Some of these approaches include intermittent preventive treatment of pregnant women (IPTp) for malaria using a bimolecular formulation of sulphadoxine – pyrimethamine combined with sleeping in insecticide treated nets (ITNs) [12-15]. These two approaches have been recommended for pregnant women living in endemic areas and are still in use today [16-18]. Effort is being made to scale – up these procedures in Cameroon.

A consensus that favored the use of these approaches was arrived at by the health authorities in Cameroon in the year 2004 and is still in force [19]. Thus, pregnant women are health-provider administered three doses of SP (three tablets per dose) during the course of pregnancy. The first dose is usually administered between the 16^th^ and the 20^th^ week or when the first fetal movements (quickening) are felt. The second dose is administered at least thirty days after the first. This interval is not fixed but is usually between the 26^th^ and the 28^th^ week of gestation and the last dose is between the 33^rd^ and the 34^th^ week. Alongside IPTp, ITNs are distributed freely to pregnant women from booking visits.

Though these procedures form a common package in our antenatal care delivery, clinical cases of malaria among pregnant women constitute about 30 to 40% of our daily hospital admissions. The aim of this study was to analyze the socio – obstetrical characteristics of cases of clinical malaria and match them against women who did not develop malaria in the same cohort.

## Methods

For a period of five years (2007–2011 inclusive), a cohort of 2525 pregnant women was followed-up from booking to delivery in the outpatient units of three tertiary level hospitals in Yaounde from where clinical cases of malaria and none malaria cases were recruited. These hospitals are all affiliated to the University of Yaounde I, Faculty of Medicine and Biomedical Sciences (FMBS). Women who were HIV infected and those who refused to participate freely in the study were not enrolled. We also eliminated women with fever producing pathologies such as acute pyelonephritis and other confirmed infections.

Enrollment was between 16 and 20 weeks of gestation. Ethical clearance N°243/CNE/SE/2006 was obtained from the National Ethics Committee and written or verbal consents were also obtained. The women had complete clinical and paraclinical evaluations. Viable pregnancies were confirmed with ultrasound. Three doses of SP were health – provider administered based on updated WHO policy recommendations. They were provided freely with ITNs on enrollment and phone calls and or home visits were carried out to ensure that the women were sleeping under ITNs.

The women were evaluated every visit for evidence of clinical malaria and those who were symptomatic for clinical malaria were hospitalized and treated. Clinical malaria was defined as an acute onset of fever, chills, anorexia, vomiting, muscular or articular pain or both and headache, all these in the absence of any concomitant pathologies such as urinary tract infection and pneumonia. Parasite densities were estimated during symptoms and compared with parasite densities from women who did not have symptoms.

### Sample size estimation

Previous studies indicated that about 30% of pregnant women develop clinical malaria in Cameroon [20]. This infers, therefore, that the proportion of pregnant women unexposed to IPTp and ITNs who are expected to exhibit the outcome of interest (Po), that is, clinical malaria is 0.3. We hypothesized that this proportion would be reduced by 50% (P1 = 0.15) if pregnant women were exposed to both methods and that no participant would leave the study (f = 0). Based on these assumptions, the required sample size for the cohort study was 133 rounded up for convenience to 166, $N\;=\;\frac{1}{1\;-\;f}\;\left(\frac{2\;\times \;\left(z_{\alpha }\;+\;z_{\beta }\right)2\;\times \;p\;\times \;\left(1\;‒\;p\right)}{\left(p_{0}\;-\;p_{1}\right)2}\right)$[21].

### Selection of exposed and unexposed cases

Cases of clinical malaria (exposed cases) and the rest of the cohort followed up were enrolled in the same period. For each case of clinical malaria enrolled, a pregnant woman recruited at the antenatal clinic who did not present with symptomatic malaria and who was matched for parity and gestational age was enrolled for comparism.

### Quantification of serum plasmodium density

Two weeks after the first dose of SP, thumb prick blood samples were collected for quantification of serum plasmodium densities which served as baseline values for the two groups. We assumed that these D1 parasite densities values would be low after treatment. Subsequently, smears were prepared from the women with clinical malaria as well as those who did not (D2 parasite densities) and compared with D1 values.

### Microscopy

Thick blood smears each measuring about 6 μL were stained with 10% Giemsa and examined under the ×100 oil immersion objective lens using Olympus CH30 microscope by two blinded laboratory scientists. Parasite densities were calculated from these thick smears and the thin smears served to differentiate Plasmodium species. The number of asexual parasites was estimated against 200 leucocytes using an average leucocytes count of 8, 000/μL as recommended [22]. A smear was considered negative only after 200 high power fields were examined.

### Estimation of parasite density

Parasite densities (parasite/μL of whole blood) were a ratio of parasites to WBCs in the Giemsa – stained thick smears. Parasites (Plasmodium specy) were counted against 200 WBCs. Calculation was as follows:

= N° of parasites counted/WBC counted × WBC count/μL of participants’s whole blood [23].

### Treatment of clinical cases

Confirmed cases were hospitalized for treatment with Quinine Sulphate at 25 mg/kg/24 hours. Three equi – doses were administered daily but not exceeding 1.5 g/24 hours irrespective of body weight or body mass index (BMI). Administration was parenteral during the first 72 hours in combination with paracetamol at 60 mg/24 hrs as adjuvant treatment. From the fourth day, both drugs were administered orally maintaining the same doses for a total duration of treatment of seven days.

### Statistical analysis

Data were entered into spread sheets and analyzed using EPI-Info™ version 3.5.1. Parasite densities were calculated based on the hypothesis that parasite densities during clinical malaria (D2) were higher than the initial densities (D1) which were considered as parasite densities before clinical malaria. We calculated odds ratios (OR) and 95% confidence intervals (C1) for the various variables we cross-tabulated that needed comparison between the exposed and unexposed cases. Kruskal-Wallis test was used to estimate the *P* – values whereby smaller *P* – values (less than 0.05) were considered statistically significant.

### Ethical considerations

Ethical clearance was obtained from the national Ethics Committee and authorization was obtained from the hospital administrations. Data collection did not require any expenditure by the women enrolled and both handling and analysis of data conformed to strict confidentiality.

## Results

A total of 2525 pregnant women were followed up during the study period out of which 166 developed clinical malaria. We selected 166 pregnant women from the same cohort who did not develop clinical malaria and compared their socio-demographic characteristics.

### Socio-demographic characteristics

An equal number of clinical cases were matched against those who did not have symptoms (166 vs 166). Greater proportions of cases were between 15 and 19 years (OR 3.42, 95% CI 2.10 – 5.59) and were of low gravidity (OR 3.77, 95% CI 2.15 – 6.61) as well as low parity (OR 2.91, 95% CI 1.68 – 5.01). The cases were single mothers (OR 4.58, 95% CI 2.54 – 8.26) and educated only at primary level (OR 3.69, 95% CI 1.58 – 4.58). Gestational ages at the time of attacks were between 20 to 30 weeks in the cases (OR 4.25, 95% CI 2.45 – 7.37). Time interval between the first and second dose of SP was greater than ten weeks in the cases (OR 5.43, 95% CI 3.04 – 9.71) (Tables 1 and 2).

**Table 1:** Demographics of participants

**Variable**	**Cohort**				
	**Malaria n = 166**	**No malaria n = 166**	**OR**	**95% CI**	**P-value**
**Age**					
*15-19*	64	36	2.097	0.55-3.41	0.036
*20-24*	24	18	1.374	1.09-4.02	0.646
*25-29*	30	25	1.275	0.69-2.35	0.533
*30-34*	20	27	0.683	0.36-1.31	0.324
*35-39*	28	40	0.299	0.30-1.37	0.339
***Level of education***					
*Higher*	16	21	0.704	0.32-1.52	0.489
*Secondary*	72	61	1.522	0.88-2.62	0.168
*Primary*	22	28	0.732	0.39-1.38	0.421
**Marital status**					
*Married*	36	33	1.127	0.56-2.24	0.771
*Single*	74	77	0.881	0.50-1.56	
**Employment status**					
*Employed*	35	27	1.434	0.79-2.59	0.294
*Unemployed*	75	83	0.697	0.39-1.26	
**Parity**					
*Primiparous*	58	30	2.974	1.70-5.22	**0.0002**
*Multiparous*	52	80	0.336	0.10-0.59	

**Table 2:** Summary of outcomes: clinical vs asymptomatic malaria

**Variables**	**Clinical malaria**	**Asymptomatic malaria**	**OR**	**95% IC**	**p**
	**(n = 116)**	**(n = 166)**			
**Maternal age (yrs)**					
**15 - 19**	68	34	5.5	3.2-9.3	0.000
**Gravidity 1 - 2**	66	28	6.5	3.8-11.3	0.000
**Parity 1 - 2**	61	32	4.6	2.7-7.9	0.000
**Primary education**	76	48	4.6	2.8-7.8	0.000
**Gestational age 20 – 30 wks**	74	34	6.8	4.1-11.7	0.000
**Gestational age > 30 wks**	3	7	0.6	0.2-2.4	0.688
**Parasite density change (%)**	64	25	6.9	3.9 - 12.1	0.000
**Time between 1**^**st**^** & 2**^**nd **^**dose**					
**10 weeks**	68	34	5.5	3.2-9.3	0.000

### Parasite densities

WBC ranged from 1,700 to 16,000 with a median of 9,300 cells/μL in clinical malaria cases and from 1,600 to 13,000 with a median of 6, 555 cells/μL in asymptomatic cases. The geometric mean of the parasites, that is SD, were 13, 234.5 and 7, 834.7 ring trophozoites/μL respectively using the actual WBC count.

## Discussion

Parasitic infections become more severe during pregnancy especially in low resource settings. Women become more susceptible to Plasmodium falciparum (PF) infection during pregnancy. The reason for this remains unclear, but changes in cell-mediated immunity are thought to be involved. Enhanced susceptibility to malaria infection means that when a woman becomes pregnant, she is most likely to develop malaria especially due to PF [24-28].

Clinical symptoms of malaria include fever, chills, sweats, nausea and vomiting [29,30]. All these symptoms were manifested by our patients. Fever occurs when schizonts rupture and release pyrogenic substances. The presence of these substances triggers the over production of cytokins such as interleukin-1 (IL-1), Interlenkin-6 (IL-6) and Tumor Necrosis Factor-alpha (TNF- alpha) [31]. These cytokins act on the thermoregulatory center of the hypothalamus, provoking a rise in the internal body temperature leading to fever, chills, shivering, nausea, headache and pain. These symptoms occur following rapid multiplication of the parasite and sequestration of infected RBCs and immune complexes along capillary walls. At this stage, the parasite density is high. We used these symptoms to define clinical malaria in our cases and we were able to demonstrate a difference in the median WBC counts and geometric mean in the two groups (Table 3). This higher parasite density during clinical malaria is responsible for the blockage or reduction of blood supply to vital organs, a situations likened to thrombotic crisis observed in sickle cell disease (SSD).

**Table 3:** Parasite densities

**Type of malaria**	**Median WBC count**	**Geometric mean**
**Clinical**	9,300 cells/μL	13, 234.5 ring trophozoites/μL
**Asymptomatic**	6, 555 cells/μL	7, 834.7 ring trophozoites/μL

The effect of P. falciparum malaria on pregnant women has been assessed in many African countries [32-34]. All the cases we had here were of P. falciparum but mixed - species Plasmodium falciparum and Plasmodium ovale malaria have been reported [35]. Results show that PF infection puts both the mother and the fetus at risk. Data from several studies show that there is a higher incidence of anaemia. Anaemia commonly occurs in women in endemic countries and becomes more severe during malaria infection. It results not only from erythrocyte destruction by the parasite, but also from the binding of malaria antigens to the surface of normal erythrocytes which are in turn removed by phagocytosis or complement mediated lyses. Evidence of an association between anaemia and malaria rather than pregnancy-associated anaemia is demonstrated by the fact that there is reduced prevalence of anaemia among pregnant women receiving antimalarial drugs for prophylaxis. Most studies show that the prevalence of malaria parasitaemia in endemic areas significantly increases during pregnancy in comparison to the same women before pregnancy and to their age-matched non-pregnant women. This is mostly marked in primigravid women, with an average 2-fold increase in prevalence compared to multigravidae [36-39].

Among the one hundred and sixty six (166) women who developed clinical malaria in this study despite IPTp and ITNs, 64 (38.55%) were between 15 and 19 years and 58 (34.94%) were in their first deliveries as against 21.69% and 18.07% respectively in asymptomatic cases. Both parity and length of gestation have an impact on susceptibility to malaria infection. The susceptibility to infection among non-immune women is similar in all parities [40]. Many arguments have been advanced to explain the increased susceptibility of pregnant women to malaria. However, the clinical manifestations depend on the degree of endemicity of infection in the local environment and the number of weeks of gestation. These largely depend on the intensity and stability of malaria transmission in the area [41].

Education plays a role as women who are not well educated are most likely to live in disadvantageous neighborhoods. A good number of women among the cases were educated at primary school level only (45.73% versus 28.92%) and gestational age during clinical malaria was between 20 – 30 weeks (45.58% versus 20.48%). The level of education did not influence significantly malaria morbidity among the cases and the controls after primary level. The occurrence of malaria among women who had attained secondary level of education was similar (OR 1.18, 95% CI, 0.67 – 2.09), to those who had university education [OR 1.06, 95% CI, 0.45 – 2.25]. One would have expected to find many women within the secondary level of education to have more episodes of clinical malaria as with primary level. However, in a country like ours where 40% of the population lives below poverty level, our neighborhoods are shared by a population mix where one finds the highly educated alongside those who have not been to school.

Malaria has always been a major public health problem in Yaounde, Cameroon where many cases of P. falciparum malaria occur every day. The prevalence of vector transmission varies from one micro-ecological area to another, resulting in variable levels of transmission [36].

Most of our patients came from the periphery of Yaounde, areas plagued with swampy waters. The bulk of our patients received the second dose of SP at intervals greater than ten weeks. This may be explained by the fact that the second dose of SP was not administered in a uniform manner by our health care providers. In some of the sites, the second dose was administered between the 26^th^ and the 28^th^ week of gestation while in some others; administration was between the 28^th^ and the 30^th^ week of gestation. Be it as it may, the interval between the first and the second dose was rather too long allowing for the effects of the drugs to fade out exposing the women to infection.

The number of cases with clinical malaria declined with increasing gestational age. The prevalence of infection was higher in the first and second trimesters of pregnancy and decreased progressively in the third trimester until delivery as described in literature [25,26]. Malaria is among the early pregnancy complications observed among pregnant adolescents in Yaounde [42].

## Conclusion

We conclude that primiparity and long intervals between the first and second dose of SP are responsible for acute bouts of clinical malaria observed among pregnant women who sleep under ITNs in our environment. Young age, low level of education and pregnancies in the first half of gestation also contribute.

## Competing interests

The authors declare that they did not have any competing interests.

## Authors’ contributions

*REM* and *HJF* conceived and designed the study, drafted the protocol, obtained ethical clearance and authorizations to conduct the study in the university affiliated hospitals. *FYF* and *FTN* worked with the hospital laboratories, analyzed data, interpreted results and wrote the various portions of the manuscript. *RT* and WAT were principal investigators respectively in two of the hospitals where the study was carried out. All the authors read and approved the final version of the manuscript and its intellectual and scientific content.

## Pre-publication history

The pre-publication history for this paper can be accessed here:

http://www.biomedcentral.com/1472-6874/14/68/prepub
